# Supplementary material for: Beyond athletic development: The effects of parkour-based versus conventional neuromuscular exercises in pre-adolescent basketball players
Source: PLoS One. 2023 Jul 12;18(7):e0288439. doi: 10.1371/journal.pone.0288439 (PMC10337934; doi:10.1371/journal.pone.0288439)
Supplement: Attachment — Submitted filename: Response to reviewers.docx [file pone.0288439.s002.docx]

Reviewer(s)’ Comments to the Author:

Reviewer: 1

| Reviewer | Response |
| --- | --- |
| It is a well written article. It brings a lot of novelty in sport sciences | We thank you and are very much appreciative of your kind words. |
| I just have minor suggestions: Please could you add the intraclass correlation coefficient (ICC) for the used test and the used type of the ICC. | Thank you for this suggestion. We have used familiarisation data for the Speed-run test and analysed this against the pre-intervention data to derive the ICC for the test. The results for the two-way mixed effects model are shown within the results section. In addition, for each test, the coefficient of variation has been included for pre- and post-intervention measures. |
| I would appreciate that authors add a table describing the characteristics of the participants (body mass, height, sitting height, maturity off set and APHV for each group). It would be nice if the authors add this characteristics before and after the intervention period. | We agreed with the requirement of this information and have included a table to display participant characteristics.  Regarding the post-intervention characteristics suggestion, we agree with that this would make a nice addition; however, we do not have post-intervention data available for the control group. We feel that due to the short-term duration of the study, which will unlikely have observed noteworthy differences relating to maturation status. |

Reviewer: 2

Comments to the Author

| Reviewer | Response |
| --- | --- |
| The proposed manuscript seems interesting and has a noteworthy objective | We would like to thank you for observing the merits of our manuscript. |
| Mixing quantitative with qualitative analysis was also interesting, however, lack of significant between-group differences does not help. | Thank you for your comments. The lack of significant differences, while we agree, are somewhat limiting, we feel that alongside the results from the thematic analysis, there are valuable outcomes that extend beyond objectively measured changes in physical performance capabilities in the child participants. We would also like to indicate the danger to science if so called “non-findings” are not identified in relation to specific training methods. The authors of many published meta-analyses readily admit that the findings of their investigations are undermined by the phenomenon of publication bias and the “file drawer problem” which occurs when the authors of perfectly rigorous and scientific studies decide not to publish their data due to not finding any significant changes in their interventions. This is a problem in sports science because it creates the false impression that specific training methods are more effective than they might be in reality because those studies that found no effect are simply disregarded and never published. On this basis, it is as important for practitioners to know what methods don’t work as it is to know what methods do work. One of the policies of journals like PLOS One is to consider the merits of rigorous scientific work which can be published regardless of the study outcome thus negating the effect of publication bias and establishing a basis for transparent science. We believe the disregarding of studies that have so called “non-findings” is anti-science and we would kindly ask the reviewer to consider such a position. |
| The fact that the intervention was only held once a week, may have not been enough to observe change. Can it be a pilot study? | We agree with your suggestion regarding the frequency of the intervention and discuss this within the manuscript though other studies have found significant improvements in response to neuromuscular training programmes performed once per week. In this population, the norm is for only one sports practice session per week. Therefore, we hope to have captured an ecological soundness to our study and feel that the work has merit beyond that of a pilot study. |
| Did the authors try to analyze the data with a General Linear Model Univariate procedure to test for any significant changes that may support the manuscript | Thank you for this comment. While it was felt that the repeated measures MANOVA was the most appropriate statistical test to run, the data was reanalysed using the GLM Univariate procedure upon your suggestion. However, this did not reveal different results to report.  We hope the mixed methods approach used in the manuscript provides further insights beyond the level of significance attained in the statistical analysis and that our discussion section satisfactorily highlights the limitations as well as presenting broader perspectives and interpretations of the results. |
