# Supplementary material for: Beyond athletic development: The effects of parkour-based versus conventional neuromuscular exercises in pre-adolescent basketball players
Source: PLoS One. 2023 Jul 12;18(7):e0288439. doi: 10.1371/journal.pone.0288439 (PMC10337934; doi:10.1371/journal.pone.0288439)
Supplement: Attachment — Submitted filename: Response to reviewers February 2023.docx [file pone.0288439.s003.docx]

Reviewer(s)’ Comments to the Author:

Reviewer: 1

| Reviewer | Response |
| --- | --- |
|  |  |
|  |  |
|  |  |

Reviewer: 2

Comments to the Author

| Reviewer | Response |
| --- | --- |
| Thank you for addressing my comments. | We would like to thank you for taking the time to review our work and your comments that have enabled us to improve the manuscript. |
| Please reconsider the conclusions reached without the necessary significance between groups. | We recognise that our conclusions required better contextualisation and have made the necessary adjustments. |
| These results cannot be extrapolated to the participants of this sport. Milder conclusions are needed to meet the lack of significant results. | Similar to our response above, we have taken your comments on board and our conclusions have been altered. |

Reviewer: 3

Comments to the Author

| Reviewer | Response |
| --- | --- |
| Overall, this is a good quality, clear, and well-written manuscript. The authors have collected noteworthy dataset and made a systematic contribution to the research literature in this area of investigation, with a strong potential for practical applications. I would like to thank the authors for their efforts in working towards improving sports’ science knowledge. I praise you for the initiative and efforts. Nevertheless, there is an area of shadow and some clarifications that have to be made in relation to the study’s statistical tool. | Firstly, we would like to thank Dr. Chelly for taking time to review our manuscript. We are most grateful for your kind words regarding our study.  We have taken on board comments regarding our statistical analysis and have made the analysis of our data more clear. To avoid ambiguity, we have removed SWC and, instead, we have displayed our results using individual participant comparisons against their own coefficient of variation. |
| The major issue with this manuscript is linked with the use of Magnitude-based inference (MBI). The author used the Hopkins’ scale (Hokins et al 2009) to compare the parkour-based, the conventional and the control groups. MBI is a controversial statistical method that has been used in hundreds of papers in sports science despite criticism from statisticians. What about the guidelines highlighted by Sainani (2018)? Analysis using the smallest worthwhile change should no more be tolerated in future sport’s science publications. | Thank you for your detailed comments. We, too, recognise that MBI and its use in sports science research is highly topical. Respectfully, however, we must point out that we did not use MBI in the analysis of our data. Instead, we used SWC which, by association to Dr. Hopkins, might have created confusion. Nevertheless, to avoid ambiguity, as mentioned in our previous response, we have removed SWC entirely from the manuscript.  We hope that, by instead providing figures that show pre-post individual changes against coefficient of variation, that we can highlight the nuanced response to training in the youth level athlete. |
